# Students and faculty perception of distance medical education outcomes in resource-constrained system during COVID-19 pandemic. A cross-sectional study

**DOI:** 10.1016/j.amsu.2021.01.073

**Published:** 2021-01-25

**Authors:** Faiz Tuma, Aussama K. Nassar, Mohamed K. Kamel, Lisa M. Knowlton, Naseer Kadhim Jawad

**Affiliations:** a Central Michigan University College of Medicine, Saginaw, USA; b Stanford University School of Medicine, Stanford, USA; c Wasit University College of Medicine, Wasit, Iraq

**Keywords:** Distance education, COVID-19, Medical education, Virtual meeting, Online learning

## Abstract

**Introduction:**

The COVID-19 pandemic has imposed significant challenges on medical education worldwide, particularly in experience- and resource-limited regions of the world. Collaborative efforts of educators and academic institutions are necessary to facilitate the adaptation to the new educational reality. In this study, challenges and outcomes of a newly implemented distance education curriculum are examined to share findings and provide recommendations.

**Methods:**

An alternative distance education curriculum with online resources and virtual lectures was developed and implemented in February 2020 at the Wasit University College of Medicine in Iraq. A post-implementation survey was developed for both faculty instructors and students to evaluate the program's effectiveness and perception. Results were compared between both groups. The study was approved by the University's Dean and exempted by the research committee for anonymity.

**Results:**

A total of 636 students and 81 instructors were surveyed. Approximately 33% of students and 51% of instructors found online education equivalent or superior to traditional face-to-face teaching methods. Almost 69% of students and 51% of instructors reported increased difficulties with virtual learning, primarily due to challenges with the available technology, unreliable internet connectivity, as well as perceive fatigue when listening to online lectures.

**Conclusions:**

Distance education provides a worthwhile alternative during the COVID-19 pandemic, including in regions of limited experience. Adequate preparation, good quality audio-visuals and Internet, and student engagement activities are recommended to improve the quality of education.

## Highlights

• Distance education is relatively new to medical education in resource-limited countries.
• This survey evaluated the feasibility of distance education and the education's perceived quality.
• Students found that online learning was difficult and required moderate technical skills.
• Instructors found the effort and time for preparation were acceptable.
• Distance education should be integrated into the standard medical education curriculum.

## 1 Introduction

On March 11, 2020, the World Health Organization (WHO) declared that the COVID-19 outbreak had reached a global pandemic level [1]. The COVID-19 pandemic significantly altered daily operations across all public sectors due to social distancing requirements. Among those affected were medical schools and universities who were challenged to adapt by providing distance medical education opportunities. Globally, medical educators have implemented major strategic shifts in their curricula and educational platforms [2,3]. Medical schools were obligated to explore “alternative methods,” including distance education and online content, and many parts of the world had to follow this path [4].

The imposed challenges created an unprecedented state of uncertainty and an unpredictable future of medical education quality and outcomes. Educational researchers and academic institutions’ efforts focused on instituting effective and applicable curricula. A few attempts to address the issues were only able to provide a limited proposal. However, medical education settings' diversity requires additional collaborative academic efforts to develop and advance the experience continuously. Collaborative efforts have been advocated to share experiences and provide solutions to the new challenging reality [5]. Schools of limited experience and or limited resources faced additional challenges.

The aim of this study is to describe, evaluate, and provide pertinent recommendations about interactive distance education in the experience and resource-limited medical schools during the COVID-19 pandemic to address the current multiple challenges. The evaluation focuses on the educational experience, outcomes, and perception of the learners and medical educators to the practice distance education. The study's framework is based on the social learning theory of Bandura and the connectivism learning theories established by George Siemens and Stephen Downes [6,7].

## 2 Method

To understand, evaluate, and discuss distance education experience in an experience- and resource-limited region during the COVID-19 pandemic, the curriculum of Wasit University College of Medicine in Iraq reviewed. Students and instructors were then asked to describe their experience with the new curriculum through a survey designed for this specific purpose. The survey questionnaire was devised by the authors and distributed electronically via mass e-mails, and responses were collected anonymously. Application of the new distance education curriculum and the users' perception were analyzed and interpreted. The study was approved by the University's Dean and exempted by the research committee for anonymity. The study was conducted in compliance with the STROCSS guidelines, and was registered in the Research Registry in accordance to Helsinki declaration (UIN: researchregistry6376. https://www.researchregistry.com/register-now#home/registrationdetails/5fdfd0b62d3a64001ca851bc/) [8].

### 2.1 Study context

Prior to the COVID-19 pandemic, Wasit University College of Medicine used face-to-face education with limited online applications. An integrated curriculum is used to provide early clinical exposure for the students. Google Classroom use was reserved for sharing learning resources, pre-assigned didactic activities, and monitoring students’ performance.

### 2.2 Curriculum changes during COVID-19 pandemic

As of February 1st, 2020, a new distance education curriculum was set in place on an urgent basis. Training on the use of virtual conferencing technology tools was provided. The basic sciences curriculum was set to contain the following components: 1) virtual lectures 3 h per day; 2) virtual small group discussions: 2 h per day; 3) bi-weekly virtual open conference; 4) bi-weekly MCQs formative assessment and discussion; and 5) bi-weekly virtual assignment presentation. Clinical sciences are taught in blocks- 13 weeks each. During the first two weeks, daily 5 h of virtual lectures are delivered plus 3 h of virtual oral assessment and discussion at the end of the two weeks. A combined virtual case discussion is conducted twice a week. Final year students have 3–5 h of virtual case presentations and discussions weekly and MCQs-based discussions twice a week. Free Conference Call software was used for the virtual synchronous sessions. While Google classroom was used for asynchronous sessions.

### 2.3 Survey questionnaire

The survey questionnaire includes ten questions for the students (Table 1) and ten questions for the teaching faculty (Table 2). Questions were developed and vetted by selected international educators, North American and local faculty, the Dean, and participating researchers of Wasit University College of Medicine. The survey was piloted among a 10% sample of faculty and trainees for readability and content. The two main domains covered in the survey were the feasibility of educational technology platforms for distance education and the education's perceived quality. The surveys were distributed securely through Survey Monkey on May 1st, 2020. Descriptive statistics were utilized to report results. Our study was exempted by the research committee of the University for anonymity.

**Table 1:** Students’ perception survey questionnaire.

1 I use online learning and participate in virtual group activities on average … … … per day: a 0–3 h, b 3–6 h, c 6–9 h, d 9–12 h, e More than 12 h, 2 What device you used mostly for online learning? a Smart phone b Tablet/iPad c Laptop d Desktop 3 What are the types of activities you participated in? a Lectures b Group discussion c Learning from the internet sources. d Asking questions/communicating with your educations. e Literature search. f Practicing solving MCQs. g Others 4 Compared to the traditional face to face learning, what do you think the overall level and quality of learning? a More b Less c- The same 5 What were the quality and clarity of audio-visual sessions and internet connectivity? a Excellent b Good c Needs improvement d Poor 6 What do you think the technical skills needed to participate and use online learning? a Very little. b Moderate. c Too much. 7 How did you find the knowledge or experience gain compared to the traditional face to face learning? a More b Less c- The same 8 The expectations and objectives of the learning activities were achieved? a Strongly disagree b Agree c Neutral d Disagree e Strongly Disagree 9 I had more difficulties in learning through online than face to face traditional learning: a Strongly disagree b Agree d Neutral e Disagree f Strongly Disagree 10 I felt tired and I lost interest from doing all the learning online: a Strongly disagree b Agree c Neutral a Disagree b Strongly Disagree

**Table 2:** **Instructors’ perception survey questionnaire**.

1 What device you used mostly for online teaching? a Smart phone. b Tablet/iPad. c Laptop. d Desktop. 2 What were the quality and clarity of audio-visual sessions and internet connectivity? a Excellent. b Good. c Fair. c Poor. d Very poor. 3 Using online teaching technology needed an acceptable amount of preparation time and effort. a Strongly disagree. b Agree. c Neutral. d Disagree. e Strongly Disagree. 4 What do you think about the technical skills needed to do online teaching? g Very little. h Moderate. i Too much. 5Using online teaching encouraged me to use technology in education. a Strongly disagree. b Agree. c Neutral. d Disagree. e Strongly Disagree. 6 How did you find the knowledge gain and effectiveness of teaching compared to the traditional face to face teaching? a Much better. b Better. b The same. c Worse. d Much worse. 7 The expectations and objectives of the teaching activities were achieved? a Strongly disagree. b Agree. c Neutral. d Disagree. e Strongly Disagree. 8 I had more difficulties in teaching through online than face to face traditional teaching: a Strongly disagree. b Agree. j Neutral. k Disagree. l Strongly Disagree. 9 I felt tired and I lost interest from doing all the teaching online: a Strongly disagree. b Agree. c Neutral. e Disagree. f Strongly Disagree. 10 Compared to the traditional face to face teaching, what do you think the overall level and quality of education? a Much better. b Better. b The same. c Worse. d Much worse.

## 3 Results

**Fig. 1:**
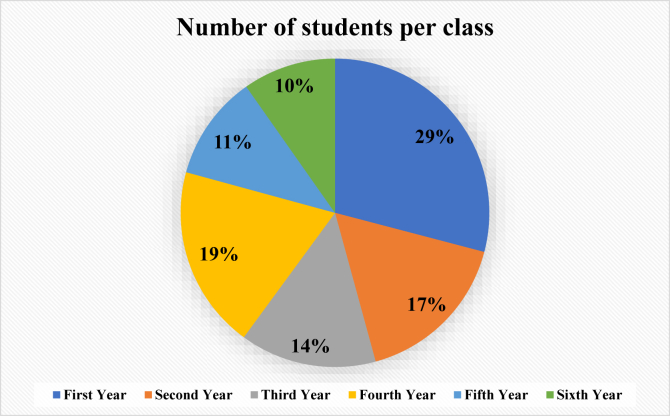
Number of students per class.

The survey-questionnaire distribution by the level of training is described in Fig. 1. Fifty-seven percent (159/280) of the respondents reported that they used their smartphone to complete online learning modules, while 31% (88/280) used a tablet or computer. Sixty-seven percent (185/278) of the students reported more difficulties with online learning than traditional learning. Twenty-seven percent of the respondents (75/278) either agreed or strongly agreed that the expectations and objectives of the online learning activities were achieved, and 67% (188/281) reported fatigue or loss of interest while participating in online learning (Table 3).

**Table 3:** Students response to the survey questionnaire.

**Q1**	I use online learning and participate in virtual group activities on average … per day:															
	0–3 h		3–6 h				6–9 h				9–12 h				>12 h	
	44%		32%				14%				5%				5%	
**Q2**	What device did you use mostly for online learning?															
	Smart phone			Tablet/iPad					Laptop					Desktop		
	57%			31%					10%					2%		
**Q3**	What are the types of activities you participated in? choose according to the frequency of use. (Score)															
	Lectures	Group discussion			Learning from internet sources			Communicating with educators		Literature search			Practice solving MCQ			Others
	5.93	4.99			4.94			4.09		3.58			3.13			1.74
**Q4**	Compared to the traditional face to face learning, what do you think the overall level and quality of learning?															
	Much more		More				The same				Less				Much less	
	7%		10%				17%				41%				25%	
**Q5**	What were the quality and clarity of audio-visual sessions and internet connectivity?															
	Excellent			Good					Needs improvement					Poor		
	2%			20%					40%					38%		
**Q6**	What do you think the technical skills needed to participate and use online learning?															
	Very little					Moderate						Too much				
	22%					56%						22%				
**Q7**	How did you find the knowledge or experience gain compared to the traditional face to face learning?															
	Much more		More				The same				Less				Much less	
	5%		10%				19%				45%				21%	
**Q8**	The expectations and objectives of the learning activities were achieved?															
	Strongly agree		Agree				Neutral				Disagree				Strongly disagree	
	2%		25%				29%				31%				13%	
**Q9**	I had more difficulties in learning online than face to face traditional learning:															
	Strongly agree		Agree				Neutral				Disagree				Strongly disagree	
	32%		37%				16%				12%				3%	
**Q1**0	I felt tired and I lost interest from doing all the learning online:															
	Strongly agree		Agree				Neutral				Disagree				Strongly disagree	
	32%		35%				18%				10%				5%	

Fifty-three percent (27/51) of the respondent instructors reported that knowledge gain and teaching effectiveness were similar or better than the traditional face to face learning, and 51% (26/51) reported that the overall level and quality of online education is similar or better compared to the traditional face to face learning. Forty-nine percent (25/51) of the respondent instructors either agreed or strongly agreed that teaching activities’ the expectations and objectives were achieved using online learning (Table 4).

**Table 4:** Instructors response to the survey questionnaire.

Q1	What device did you use mostly for online teaching													
	Smart phone			Tablet/iPad				Laptop				Desktop		
	32%			3.5%				57%				7.5%		
**Q2**	What were the quality and clarity of audio-visual sessions and internet connectivity?													
	Excellent		Good				Fair			Poor			Very poor	
	16%		38%				36%			10%			0%	
**Q3**	Using online teaching technology needed an acceptable amount of preparation time and effort?													
	Strongly agree		Agree				Neutral			Disagree			Strongly disagree	
	25%		63%				6%			4%			2%	
**Q4**	What do you think the technical skills needed to participate and use online learning?													
	Very little				Moderate						Too much			
	6%				76%						18%			
**Q5**	Using online teaching encouraged me to use technology in education?													
	Strongly agree		Agree				Neutral			Disagree			Strongly disagree	
	23%		59%				10%			6%			2%	
**Q6**	How did you find the knowledge gain and teaching effectiveness compared to the traditional face to face learning?													
	Much better		Better				The same			Worse			Much worse	
	6%		27%				20%			43%			4%	
**Q7**	The expectations and objectives of the teaching activities were achieved?													
	Strongly agree	Agree				Neutral			Disagree					Strongly disagree
	12%	37%				37%			12%					2%
**Q8**	I had more difficulties in teaching online than face to face traditional teaching:													
	Strongly agree	Agree				Neutral			Disagree					Strongly disagree
	14%	37%				27%			20%					2%
**Q9**	I felt tired and I lost interest from doing all the teaching online:													
	Strongly agree	Agree				Neutral			Disagree					Strongly disagree
	8%	27%				22%			39%					4%
**Q1**0	Compared to the traditional face to face learning, what do you think the overall level and quality of education?													
	Much better		Better				The same			Worse			Much worse	
	4%		16%				31%			45%			4%	

## 4 Discussion

The experience of distance education or online learning is relatively new to medical education in Iraq [[9], [10], [11]]. There have been few sporadic attempts to use technology in learning from a distance [11]. These attempts were limited in terms of the duration of their implementation, their scope, and their objectives. Prior research identified barriers to distance education in the pre COVID19 era, which were the limited availability of the academic support, resources, and long-term plans as well as the limited official data about the current status of distance education application in the academic institutions [12]. Addressing these barriers swiftly from different aspects by multi-disciplinary groups was crucial to successfully implementing distance education.

### 4.1 Feasibility and challenges of distance education platform

Students found that online learning was difficult and required moderate technical skills compared to face-to-face learning. By comparison, instructors found the effort and time for preparation were acceptable, and they did not feel more online teaching difficulties than face-to-face. Students appreciate online learning than face to face [13]. However, other factors influence the overall experience. Example of these factors are like poor instructional design, sub-optimal or poor Internet connection or audio-visual media quality, or unfamiliarity to the complete online learning suddenly came in effect. Non-academic and sports activities attract students to in-person education.

Instructors found the effort and time for preparation are acceptable. They were encouraged to use technology in education after this experience. While instructors need more work and continuous improvement and motivation to teach online [14], they become more efficient once they are more familiar with the curriculum's setup and operation. The experience's context and educational process have influenced the instructors' perception of challenges and difficulty accepting new realism. Therefore, distance education instructors should aim for the long-term benefits and efficiency that outbalance the initial challenges.

The audio-visual streaming experience's quality and clarity, mostly reflective of internet connection, were low as most students perceived but were fair-good for the instructors and teaching faculty. This is a significant finding of the study. The impact of the Internet in education is a well-recognized concept in education research [15]. For distant synchronous education, efficient bandwidth and speed Internet is essential for effective education.

Studies of the devices used by students in higher education online learning showed a lower rate of smartphone use versus laptops or tablets, which might be affected by demographic and socio-economic factors related to device access [16]. This is a convenience and practicality choice. It could also reflect the affordability of smartphones by students with limited income. Therefore, no additional devices are deemed necessary or prerequisites to use distance education.

### 4.2 Quality of educational process and outcomes

Most students felt that meeting expectations and objectives were either equivocal or non-achieved, while instructors were more agreeing with meeting the objectives. Contrary to the student's perception, instructors found knowledge gain and effectiveness of online education's educational experience is the same as in face-to-face education. The debate of distance education effectiveness compared to face-to-face education is an old, long, and continuous debate, with several studies demonstrate the benefits of distance learning [[17], [18], [19]]. There is a wide variability of the specific purpose of using distance learning. This reflects on the value of the utility. In terms of educational materials and resources, the online style's advantages are numerous [17]. For social interaction and learning under the social constructivism theory, distance learning is not as effective as the face-to-face education. Students preferred face-to-face learning for communication opportunities where they share understanding and learning through interacting [13,20]. The balance among the various aspects and priorities has to be carefully considered by educators and programs to achieve the educational process's ultimate goals.

The students and instructors also evaluated the overall quality of the educational experience. Most instructors rated the overall quality as the same or worse than the face-to-face education. This rating is slightly better than the students’ rating. Most students found that the overall level and quality of distance learning is less than face-to-face. On evaluating the motivation to continue the current online approach if the circumstances of the pandemic mandate, most instructors showed interest and did not feel tired by online teaching. In contrast, most students felt overwhelmed and lost interest in virtual learning. This is a significant factor that should be considered if continuing online education plans are to be continued. Educators have to address the difficulties faced by the students and provide an optimal studying environment and setup.

### 4.3 Limitations and important considerations

The study results and the description of distance education are aimed to provide an overview of the experience to the local, regional, and international educators and institutions. Many findings and recommendations have been concluded. However, it is essential to realize that the study does not evaluate the standard distance or online education or compares distance to face-to-face education. Distance learning was used as an alternative option, on an urgent basis, for every aspect of the learning, and without adequate planning and preparation. The key lesson to be learned from this experience is that distance learning outcomes used on an urgent basis differ from those used with adequate prerequisites. The study presents results and evaluation of the experience that reflect a cross-sectional examination of the distance education experience applied on-the-go basis. Qualitative evaluation of the experience through interviews or focus group discussions (FGD) would add in-depth understanding and evaluation in an explanatory or exploratory sequential fashion [21]. Interviews or FGD facilitate exploring the meaning of findings and results of surveys that are difficult to explain statistically, reveal a range of opinions and views on a particular topic of interest or collect a wide variety of local factors [22].

## 5 Conclusion

The study reflects an evaluation and review of distance medical education curriculum implementation due to COVID-19 with limited prior experience and preparedness. Although users perceived the distance education format as less effective, they found it a worthwhile alternative to the traditional face-to-face education during the pandemic. Improving the educational, cultural, and technical challenges augments better outcomes and widespread adoption. Adequate prior planning and incorporating engaging activities for the students boost the quality of education. Further opportunities for improvement and implementation strategies should be characterized through qualitative or mixed methods studies to provide well-structured actionable next steps for gradually integrating distance education into the standard medical education curriculum in the foreseeable future after the pandemic.

## Funding

None.

## Ethical approval

Ethical approval was obtained by University of Wasit/College of Medicine Ethical Committee.

## Consent

No written consent was required as the study did not include patients.

## Author contribution

Study concept or design: Faiz Tuma, Aussama K. Nassar, Mohamed K. Kamel, Lisa M. Knowlton, Naseer Kadhim Jawad.

Data collection: Faiz Tuma, Aussama K. Nassar, Mohamed K. Kamel, Lisa M. Knowlton, Naseer Kadhim Jawad.

Data analysis or interpreta Faiz Tuma, Aussama K. Nassar, Mohamed K. Kamel, Lisa M. Knowlton, Naseer Kadhim Jawad.tion:

Writing the paper: Faiz Tuma, Aussama K. Nassar, Mohamed K. Kamel, Lisa M. Knowlton, Naseer Kadhim Jawad.

## Registration of research studies

1. Name of the registry: Research Registry.

2. Unique Identifying number or registration ID: researchregistry6376.

3. Hyperlink to your specific registration (must be publicly accessible and will be checked): https://www.researchregistry.com/register-now#home/registrationdetails/5fdfd0b62d3a64001ca851bc/

## Guarantor

Faiz Tuma, MD.

## Declaration of competing interest

None.
